# Cloning, Characterization and Heterologous Expression of the Indolocarbazole Biosynthetic Gene Cluster from Marine-Derived *Streptomyces sanyensis* FMA

**DOI:** 10.3390/md11020466

**Published:** 2013-02-06

**Authors:** Tong Li, Yuanyuan Du, Qiu Cui, Jingtao Zhang, Weiming Zhu, Kui Hong, Wenli Li

**Affiliations:** 1 Key Laboratory of Marine Drugs, Ministry of Education of China, School of Medicine and Pharmacy, Ocean University of China, Qingdao 266003, China; E-Mails: oaixlittle@163.com (T.L.); awetobean@126.com (Y.D.); weimingzhu@ouc.edu.cn (W.Z.); 2 Qingdao Institute of Bioenergy and Bioprocess Technology, Chinese Academy of Sciences, No. 189 Songling Road, Qingdao 266101, China; E-Mails: cuiqiu@qibebt.ac.cn (Q.C.); zhang_jt@qibebt.ac.cn (J.Z.); 3 Ministry of Education of China, School of Pharmaceutical Sciences, Wuhan University, Wuhan 430071, China

**Keywords:** indolocarbazole (ICZ), biosynthesis, heterologous expression, *Streptomyces sanyensis* FMA

## Abstract

The indolocarbazole (ICZ) alkaloids have attracted much attention due to their unique structures and potential therapeutic applications. A series of ICZs were recently isolated and identified from a marine-derived actinomycete strain, *Streptomyces sanyensis* FMA. To elucidate the biosynthetic machinery associated with ICZs production in *S.* *sanyensis* FMA, PCR using degenerate primers was carried out to clone the FAD-dependent monooxygenase gene fragment for ICZ ring formation, which was used as a probe to isolate the 34.6-kb DNA region containing the *spc* gene cluster. Sequence analysis revealed genes for ICZ ring formation (*spcO*, *D*, *P*, *C*), sugar unit formation (*spcA*, *B*, *E*, *K*, *J*, *I*), glycosylation (*spcN*, *G*), methylation (*spcMA*, *MB*), as well as regulation (*spcR*). Their involvement in ICZ biosynthesis was confirmed by gene inactivation and heterologous expression in *Streptomyces coelicolor* M1152. This work represents the first cloning and characterization of an ICZ gene cluster isolated from a marine-derived actinomycete strain and would be helpful for thoroughly understanding the biosynthetic mechanism of ICZ glycosides.

## 1. Introduction

**Scheme 1:**
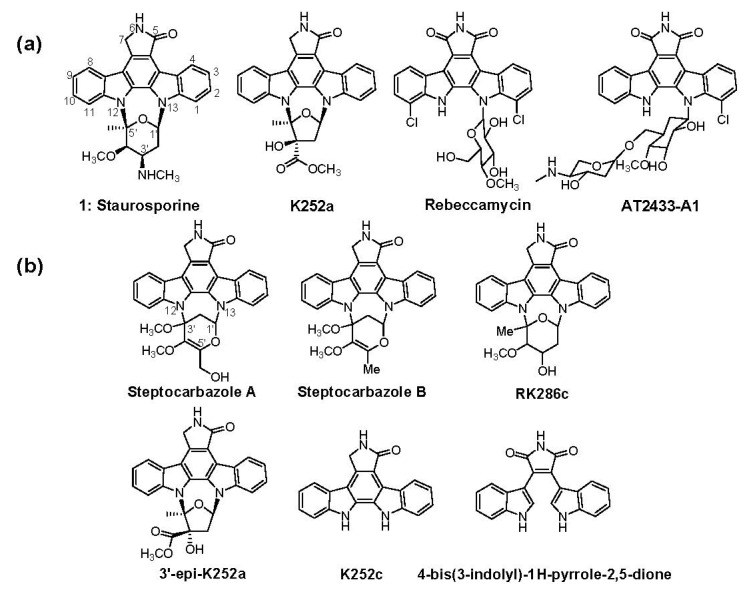
(**a**) Structures of staurosporine (STA, **1**), K252a, rebeccamycin and AT2433-A1; (**b**) Structures of indolocarbazoles (ICZs) isolated from *S. sanyensis* FMA besides STA and K252a.

The firstly reported indolocarbazole (ICZ) was staurosporine (STA, **1**), which was isolated from the fermentation culture of *Streptomyces staurosporeus* AM-2282 (ATCC 55006) in 1977 (Scheme 1) [1]. Since then, more than 130 ICZs have been isolated from various organisms, including bacteria, fungi and invertebrates, during the last 35 years [2,3,4]. Due to their unique structures and important biological bioactivities, this family of compounds has attracted much attention ever since its discovery. ICZs have been found to inhibit protein kinase, topoisomerase and ATP-binding cassette transporter [2,5,6,7,8,9]. Several ICZs, such as UCN-01 (7-hydroxy-STA), lestaurtinib (CEP-701), midostaurin (PKC412), edotecarin, becatecarin and NSC655649, are presently undergoing clinical trials for novel antitumor therapies [10]. 

**Figure 1:**
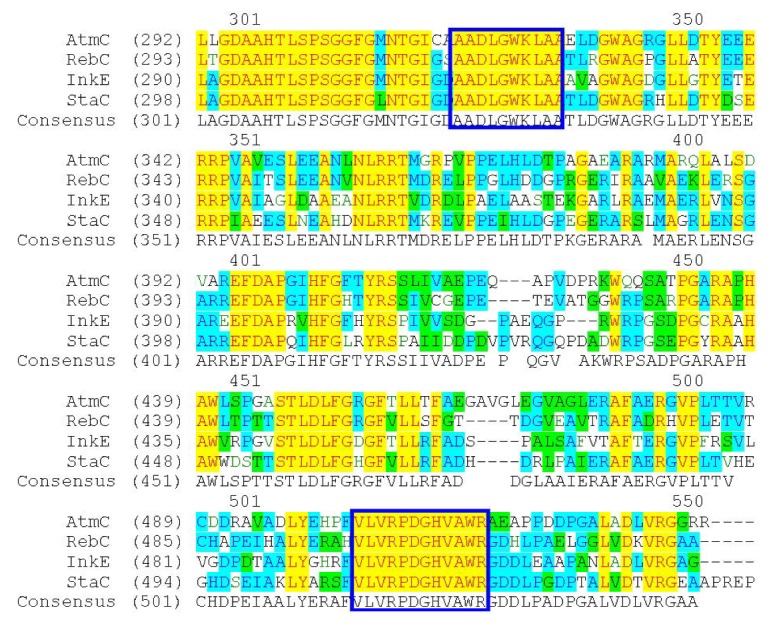
Alignment of AtmC (ABC02791), RebC (CAC93716), InkE (ABD59214) and StaC (BAF47693). The conserved regions of amino acids for degenerate primers design are indicated in the squares.

The therapeutic diversity and medicinal potential of ICZs have inspired interest in their biosynthesis research. The biosynthetic gene clusters for rebeccamycin (REB), STA, AT2433-A1 and K252a (Scheme 1) have been reported [11,12,13,14,15], and their assembling mechanisms have been widely studied [2,16,17,18,19,20,21]. REB and AT2433-A1 are halogenated and contain a fully oxidized C-7 carbon and a β-glycosidic bond to only one indole nitrogen; conversely, STA and K252a are not halogenated and bear a fully reduced C-7 carbon and a sugar attached to both indole nitrogens (Figure 1). The ICZ rings are synthesized from two molecules of tryptophan involving a series of oxidation steps. These reactions include amino oxidation (catalyzed by amino oxidases RebO/StaO/AtmO/InkO/NokA), chromopyrrolic acid formation (catalyzed by heme-dependent oxidases RebD/StaD/AtmD/InkD/NokB), ring-closing reaction (catalyzed by cytochrome P450s RebP/StaP/AtmP/InkP/NokC) and oxidative decarboxylation (catalyzed by FAD-dependent monooxygenases RebC/StaC/AtmC/InkE/NokD) [16,19,20,22,23,24]. As a result, two classes of aglycone scaffolds—arcyriaflavin A (for REB and AT2433-A1) and K252C (for STA and K252a), are generated, respectively.

In recent years, an increasing amount of ICZs were isolated from marine-derived strains; in addition, the isolated ICZs are usually comprised of multiple analogs [4,25,26,27]. However, genetic information regarding these compounds has been seldom reported. Recently, a series of ICZs were isolated and identified from *S . sanyensis* FMA(=219808), which was isolated from mangrove soil samples collected in Sanya, Hainan Province of China [28]. The characterized ICZs include K252c, K252a, 3′-epi-K252a, RK286c, 4-bis(3-indolyl)-1*H*-pyrrole-2,5-dione and two novel ICZs, streptocarbazoles A and B (Scheme 1). The bioassay experiments revealed that streptocarbazole A was cytotoxic on HL-60 and A-549 cell lines and could arrest the cell cycle of Hela cells at the G_2_/M phase [4]. In contrast to all the other reported cyclic ICZ glycosides, which typically bear cyclic *N*-glycosidic linkages between the 1,5-carbons of the glycosyl moiety and two indole nitrogens of K252c, the aglycones of streptocarbazoles A and B are linked to the 1,3-carbons of the glycosyl moiety, indicating that a novel enzymatic mechanism might be involved in the C–N bond formation between the C-3′ of deoxysugar and the *N*-12 of aglycone (Scheme 1). This exceptional cyclic *N*-glycosidic linkage prompted us to investigate the biosynthetic mechanism of ICZs compounds in the marine-derived *S. sanyensis* FMA. Therefore, we did alignments of the ICZ ring biosynthetic genes and designed a pair of degenerate primers to amplify the corresponding DNA fragment encoding the FAD-dependent monooxygenase from strain FMA. A cosmid library of strain FMA was constructed, from which the *spc* biosynthetic gene cluster encoding ICZ production was isolated, which was surprisingly highly homologous to the STA gene cluster from *Streptomyces* sp. TP-A0274. Here, we report the cloning, characterization and heterologous expression of the *spc* gene cluster from *S. sanyensis* FMA. 

## 2. Results and Discussion

### 2.1. Cloning and Sequencing of the *spc* Gene Cluster from *S. sanyensis* FMA

The formation of ICZ rings involves four conserved enzymes; therefore, a pair of degenerate primers were designed according to the alignment result of RebC (CAC93716), StaC (BAF47693), AtmC (ABC02791) and InkE (ABD59214), which revealed highly conserved regions of AADLGWKLAA and VLVRPDGHVAWR, as shown in Figure 1. A distinct product at the expected size of 0.6 kb was obtained by PCR from genomic DNA of the strain FMA using the degenerate primers and was cloned into pUM-T to yield pWLI601. Sequencing results showed that the PCR-amplified product was very similar to known FAD-dependent monooxygenases for ICZ ring biosynthesis, with 59% identity to RebC, 71% identity to StaC, 59% identity to AtmC and 55% identity to InkE, indicating that the amplified gene fragment is probably involved in ICZ biosynthesis in strain FMA. This cloning strategy would be applicable for probing the biosynthetic genes for other ICZ ring-containing natural products.

The cosmid library of strain FMA was constructed using SuperCos1 as the vector. A pair of specific primers was designed according to the internal sequence of the 0.6 kb fragment and was used for library screening. Five overlapped positive cosmids (pWLI611-615) were identified, and pWLI615 was chosen for further sequencing, ultimately giving a 34.6 kb continuous DNA region. The overall G + C content of the region was 75.5%. The sequence was deposited in the GenBank database under the accession number KC182794. 

### 2.2. Organization and Characterization of the *spc* Gene Cluster

**Figure 2:**
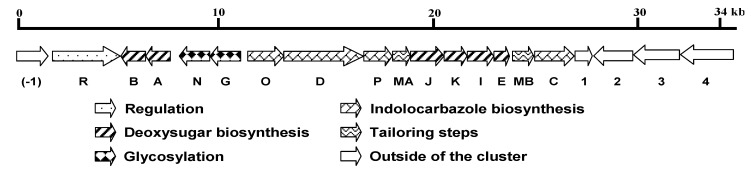
Genetic organization of the *spc* biosynthetic gene cluster. Proposed functions of individual open reading frames are coded with various patterns and summarized in Table 1.

In total, 19 open reading frames (ORFs) were identified, among which 15 were designated as ICZ glycoside biosynthetic genes and the other four were predicted to be beyond the cluster (Figure 2). The composition and organization of the cluster are highly conserved with the STA gene cluster from *Streptomyces* sp. TP-A0274. The results are summarized in Table 1. *spcODPC* genes, which exhibit 65%–78% identity to the known homologous genes, encode the ICZ ring K252c. *spcABEKJI* genes, showing 64%–86% identity to their homologs, are responsible for the assembly of the sugar moiety, followed by C–N bond formation, catalyzed by SpcG and SpcN, sequentially. The two methylation-tailoring steps are performed by SpcMA and SpcMB, respectively. Expression of the gene cluster is probably regulated by SpcR, a LuxR family transcriptional activator harboring a typical Helix-Turn-Helix (HTH) motif for DNA binding at the *C*-terminus. 

**Table 1:** Proposed functions of proteins encoded by the *spc* biosynthetic gene cluster in *S. sanyensis* FMA andits comparison with the STA gene cluster in *S treptomyces* sp. TP-A0274.

Protein	Size (aa)	Proposed function	Homolog in strain TP-A0274 (Accession No.)	% Identity/Similarity
Orf(-1)	341	hypothetical protein	-	-
SpcR	986	transcriptional regulator	StaR (BAC55205.1)	62/70%
SpcB	357	dTDP-glucose 4,6-dehydratase	StaB (BAC55206.1)	82/87%
SpcA	354	glucose-1-phosphate thymidyltransferase	StaA (BAC55207.1)	78/87%
SpcN	390	cytochrome P450	StaN (BAC55208.1)	76/81%
SpcG	433	*N*-glycosyltransferase	StaG (BAC55209.1)	76/84%
SpcO	503	L-amino acid oxidase	StaO (BAC55210.1)	77/85%
SpcD	1123	chromopyrrolic acid synthase	StaD (BAC55211.1)	65/71%
SpcP	427	cytochrome P450	StaP (BAC55212.1)	71/79%
SpcMA	277	methyltransferase	StaMA (BAC55213.1)	63/72%
SpcJ	477	2,3-dehydratase	StaJ (BAC55214.1)	64/70%
SpcK	331	4-ketoreductase	StaK (BAC55215.1)	75/83%
SpcI	369	aminotransferase	StaI (BAC55216.1)	86/92%
SpcE	208	3,5-epimerase	StaE (BAC55217.1)	83/89%
SpcMB	281	methyltransferase	StaMB (BAC55218.1)	75/83%
SpcC	542	monooxygenase	StaC (BAF47693.1)	78/86%
Orf1	238	hypothetical protein	-	-
Orf2	540	integral membrane protein	-	-
Orf3	292	outer membrane adhesion like protein	-	-
Orf4	660	integral membrane protein	-	-
Orf5	884	D-alanyl-D-alanine carboxypeptidase	-	-

-: means not available.

**Figure 3:**
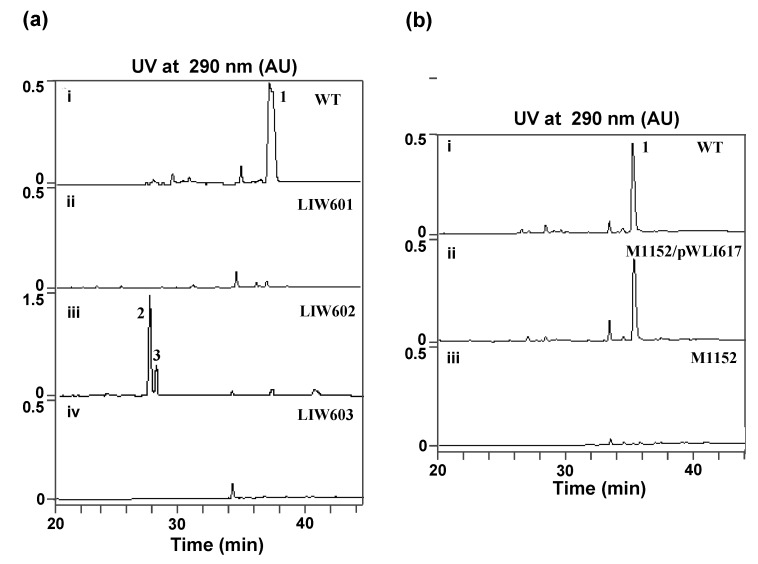
(**a**) HPLC traces of fermentation products of *S. sanyensis* FMA strains; (**b**) HPLC traces of fermentation products of *S. coelicolor* M1152 strains. (a): (i) Wild-type strain FMA; (ii) *spcC* mutant LIW601; (iii) *spcI* mutant LIW602; (iv) *spcR* mutant LIW603. (b): (i) Wild-type strain FMA; (ii) *S. coelicolor* M1152/pWLI617; (iii) *S. coelicolor* M1152. The ICZs compounds STA (1), K252d (2) and K252c (3) are indicated.

Although 7 ICZs—K252c, K252a, 3′-epi-K252a, RK286c, 4-bis(3-indolyl)-1*H*-pyrrole-2,5-dione and streptocarbazoles A and B compounds have been originally isolated from *S. sanyensis* FMA, to our surprise, the isolated ICZ gene cluster exhibit high homology to that of the STA gene cluster from *Streptomyces* sp. TP-A0274. Further fermentation of strain FMA showed the major ICZ compound accumulated in strain FMA is indeed STA (Figure 3). We assume that the backbone formation of the previously isolated ICZs is directed by the *spc* gene cluster, and certain enzyme(s) beyond the cluster might be involved in the biosynthesis of some minor ICZ components as well. Culture conditions may influence gene expression, leading to metabolic changes and, consequently, resulting in the different metabolite profile of ICZs in this strain. Additional 20 kb DNA regions both upstream and downstream of the defined gene cluster were further analyzed for possible genes involving biosynthesis of the minor ICZs components (data not shown). No obvious hit was obtained. In addition, genome sequencing has been performed [29], revealing that the *spc* gene cluster is the only ICZ biosynthesis locus in the genome, and further analysis is currently going on. 

### 2.3. Involvement of the *spc* Gene Cluster in ICZs Biosynthesis in *S. sanyensis* FMA

**Figure S1:**
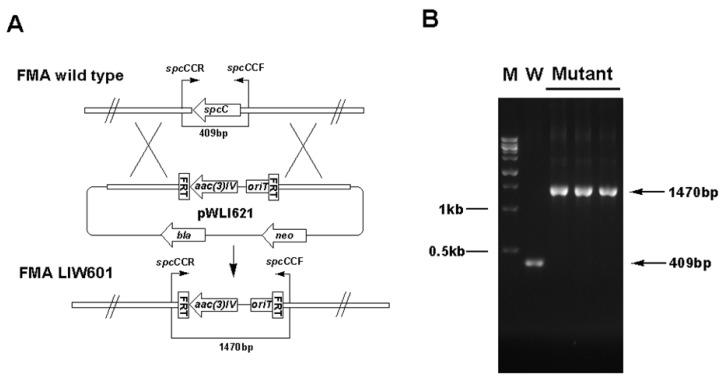
Inactivation of *spcC*. (**A**) Construction of *spcC* gene inactivation mutant. (**B**) PCR confirmation of the double-crossover mutant. M: DNA marker; W: *S. sanyensis* FMA wild type strain; Mutant: *spcC* gene inactivation mutant.

**Figure S2:**
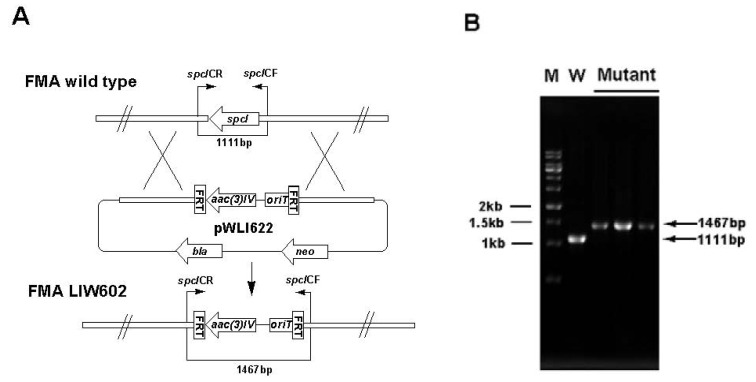
Inactivation of *spcI*. (**A**) Construction of *spcI* gene inactivation mutant. (**B**) PCR confirmation of the double-crossover mutant. M: DNA marker; W: *S. sanyensis* FMA wild type strain; Mutant: *spcI* gene inactivation mutant.

**Figure S3:**
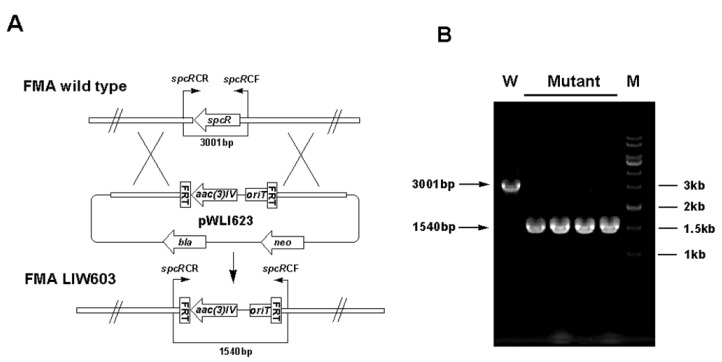
Inactivation of *spcR*. (**A**) Construction of *spcR* gene inactivation mutant. (**B**) PCR confirmation of the double-crossover mutant. M: DNA marker; W: *S. sanyensis* FMA wild type strain; Mutant: *spcR* gene inactivation mutant.

To support the predicted involvement of this locus in the ICZs biosynthesis in *S. sanyensis* FMA, *spcCIR* genes were inactivated by using the PCR targeting strategy (Table S2, Supplementary Materials). The target genes were replaced with the *aac(3)IV/oriT* cassette, resulting in mutant cosmids pWLI621 (Δ*spcC*), pWLI622 (Δ*spcI*) and pWLI623 (Δ*spcR*), which were then transferred into the wild type *S. sanyensis* FMA. Apramycin-resistant (Apr^R^) and kanamycin-sensitive (Kan^S^) exconjugants were selected as double crossover mutants. LIW601 (Δ*spcC*), LIW602 (Δ*spcI*) and LIW603 (Δ*spcR*) and their genotypes were confirmed by PCR (Figure S1, Figure S2, Figure S3, Supplementary Materials). All the mutants were fermented and tested for ICZs formation, using the wild-type strain as a positive control. Inactivation of *spcC* almost completely abolished ICZs production in LIW601 (Figure 3A, panel ii), which proved its essential role for ICZs biosynthesis, consistent with previously reported results [19,23]. No ICZs production was observed in LIW603, indicating that *spcR* is a positive regulator (Figure 3a, panel iv). Conversely, *spcI* mutant accumulated two compounds (**2** and **3**) with very similar UV-vis spectra to that of STA (Figure 3a, panel iii). 

**Scheme 2:**
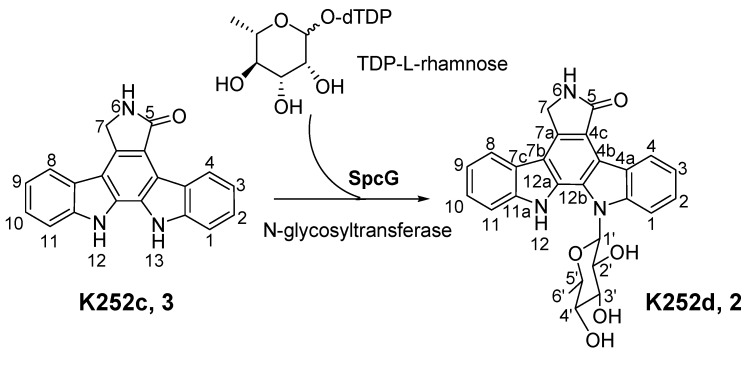
Proposed formation of K252d in the *ΔspcI* mutant LIW602.

**Figure S4:**
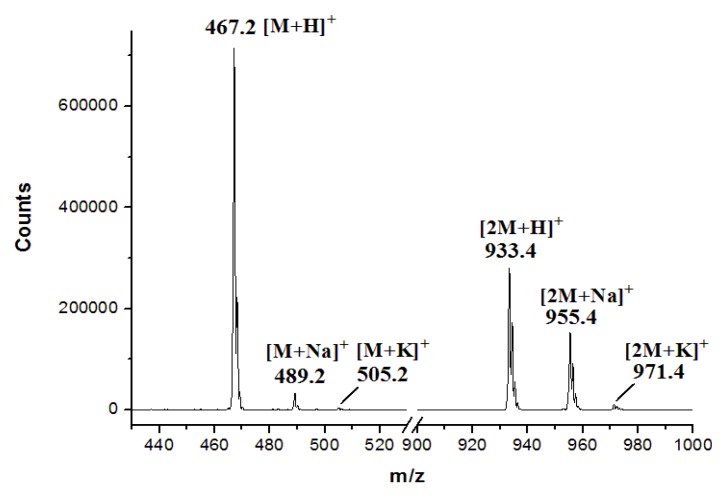
ESI-MS spectrum of STA.

**Figure S5:**
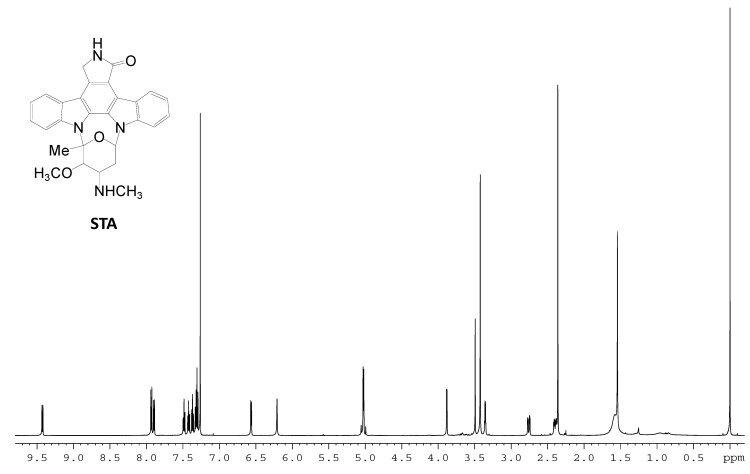
^1^H NMR spectrum of STA.

**Figure S6:**
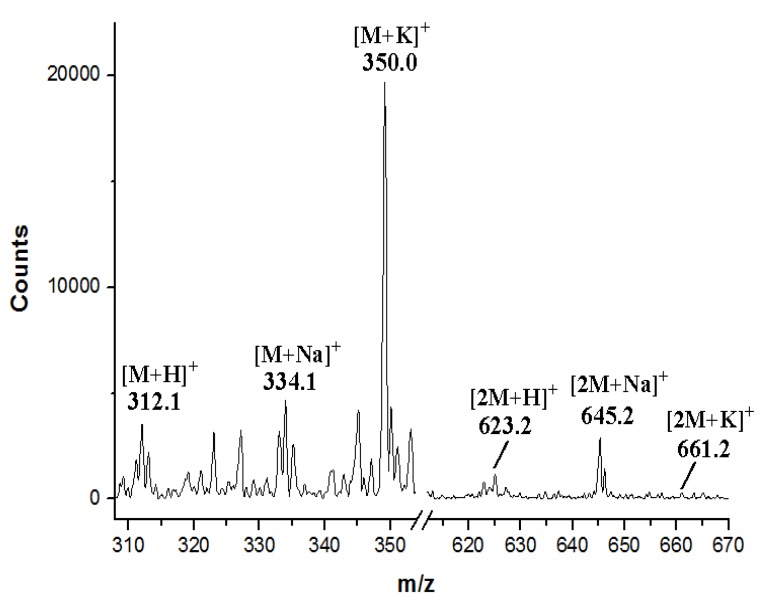
ESI-MS spectrum of K252c.

**Figure S7:**
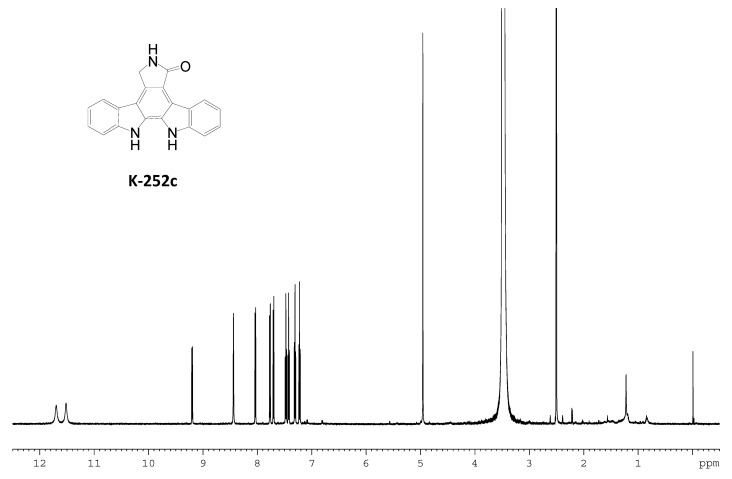
^1^H NMR spectrum of K-252c.

**Figure S8:**
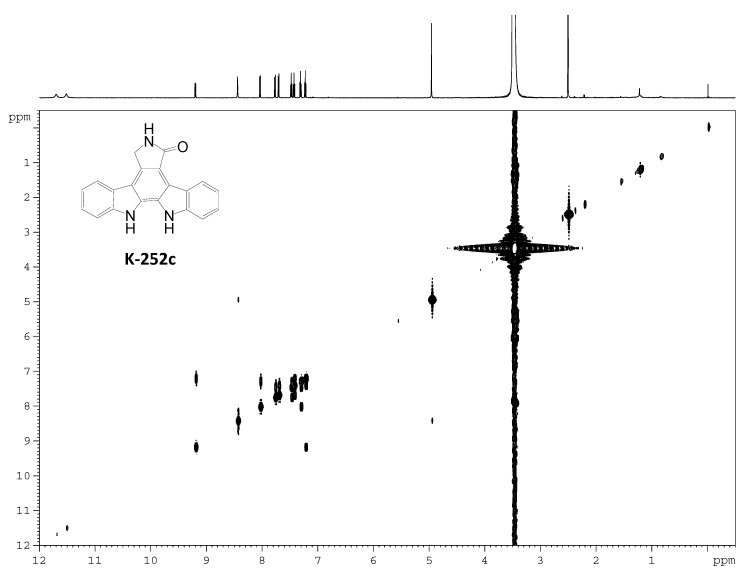
^1^H–^1^H COSY spectrum of K-252c.

**Figure S9:**
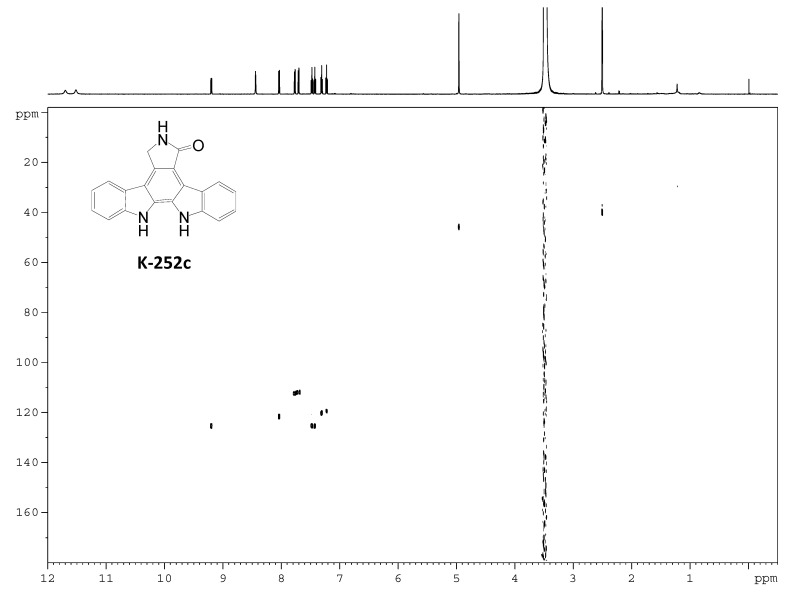
HSQC spectrum of K-252c.

**Figure S10:**
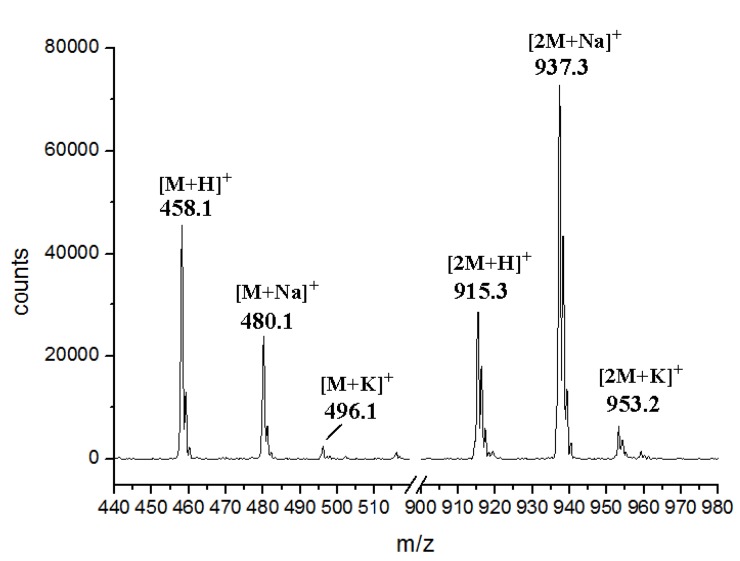
ESI-MS spectrum of K-252d.

**Figure S11:**
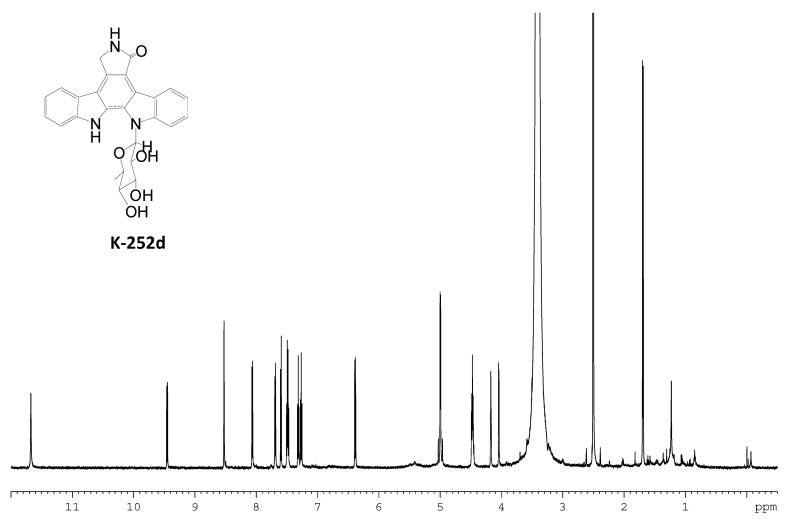
^1^H NMR spectrum of K-252d.

**Figure S12:**
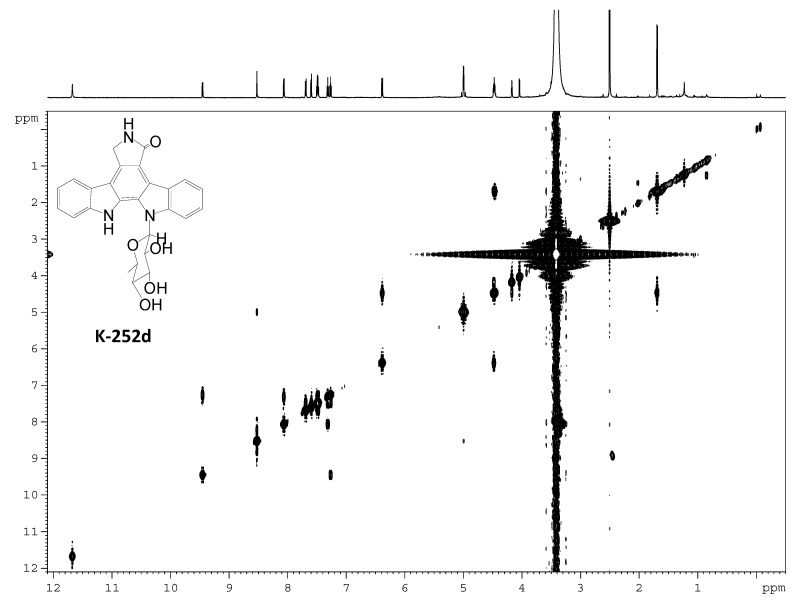
^1^H–^1^H COSY spectrum of K-252d.

**Figure S13:**
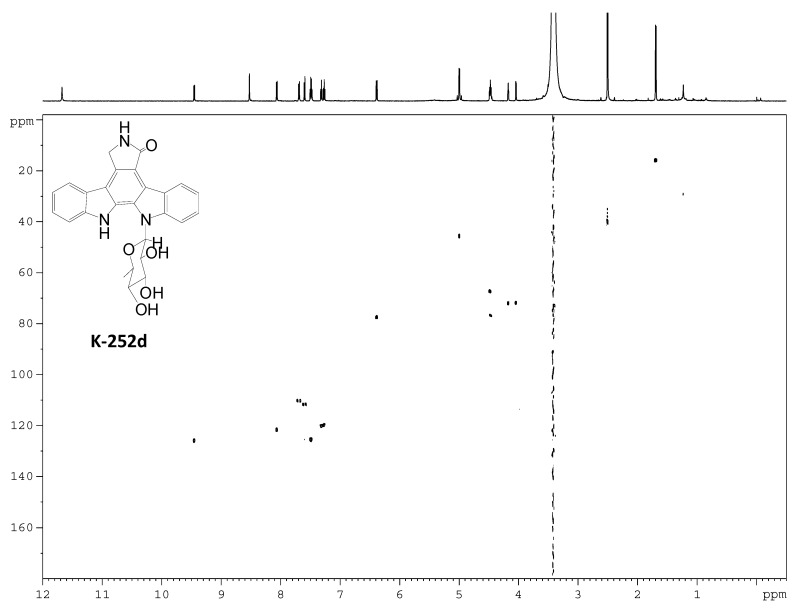
HSQC spectrum of K-252d.

**Figure S14:**
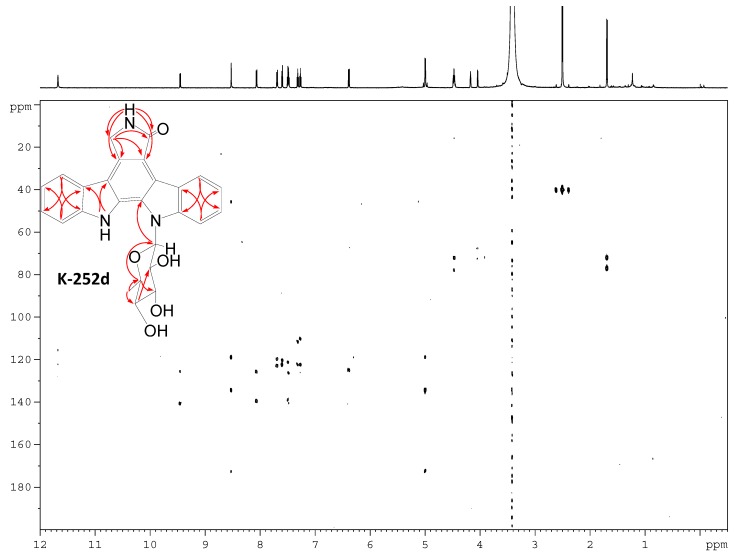
HMBC spectrum of K-252d.

The identity of the predominant ICZ compound, STA, produced by strain FMA was confirmed by MS and ^1^H NMR analysis, which were identical to those previously reported [30] (Figure S4 and Figure S5, Supplementary Materials). The two compounds produced by *spcI* mutant were isolated, and their structures were determined by MS, ^1^H NMR, ^13^C NMR, ^1^H–^1^H COSY, HSQC and HMBC (for two) data (Figure S6, Figure S7, Figure S8, Figure S9, Figure S10, Figure S11, Figure S12, Figure S13, Figure S14, Supplementary Materials). Compound **2** was identified as K252d, with a molecular weight of 457, while compound **3** is K252c, with a molecular weight of 311. Interestingly, only K252c was reported to be accumulated in the* ΔstaI* mutant of *Streptomyces* sp. TP-A0274 [31]. Therefore, we proposed that inactivation of *spcI* led to accumulation of both TDP-sugar and ICZ ring, and further, *spcG* may use TDP-L-rhamnose as a sugar donor to catalyze its attachment onto the *N*-13 atom of K252c to afford K252d (Scheme 2). The substrate promiscuity of SpcG would make it an alternative tool for glycodiversification. 

### 2.4. Heterologous Expression of the *spc* Gene Cluster in *Streptomyces coelicolor* M1152

Heterologous expression was performed in *S. coelicolor* M1152, which is well characterized and does not harbor ICZs gene clusters in its genome. pWLI615 was equipped with *oriT* and φC31 attP/int for conjugation and integration at the *attB* site at the chromosome, resulting in pWLI617. To test production in a heterologous host, pWLI617 was introduced into *S. coelicolor* M1152 by conjugation. Apramycin-resistant exconjugants were selected to generate *S. coelicolor* M1152/pWLI617. HPLC analysis of the fermentation cultures showed that *S. coelicolor* M1152/pWLI617 produced STA in an excellent yield, in contrast, STA was completely absent from *S. coelicolor* M1152 (Figure 3b). The identity of STA was confirmed by MS analysis, giving the characteristic molecular ions (*m/z* for [M + H]^+^ of 467.2), consistent with the theoretical calculated molecular mass for C_28_H_26_N_4_O_3_ (Figure S4, Supplementary Materials). Thus, the *spc* gene cluster was successfully expressed in the heterologous host, demonstrating its integrity for STA biosynthesis. 

## 3. Experimental Section

### 3.1. Bacterial Strains, Plasmids and Reagents

Bacterial strains and plasmids used and constructed during this study are listed in Table S1. *Escherichia coli* DH5α was used as the host for general subcloning [32]. *E. coli* Top10 (Invitrogen, Carlsbad, La Jolla, CA, USA) was used as the transduction host for cosmid library construction. *E. coli* ET12567/pUZ8002 [33] was used as the cosmid donor host for *E. coli-Streptomyces* intergeneric conjugation. *E. coli* BW25113/pIJ790 was used for λRED-mediated PCR-targeting [34]. *S. sanyensis* FMA wild-type strain has been described previously [4,28]. *E. coli* strains were grown and manipulated following standard protocols [32,34,35]. *S. sanyensis* FMA strains were grown at 30 °C on ISP-4 medium for sporulation and conjugation and were cultured in TSB medium for genomic DNA preparation. Common biochemicals and chemicals were purchased from standard commercial sources.

### 3.2. DNA Manipulation, Sequencing and Bioinformatic Analysis

Plasmid extractions and DNA purification were carried out using commercial kits (OMEGA, BIO-TEK). Genomic DNAs were prepared according to the literature protocol [36]. Both primer synthesis and DNA sequencing were performed at Sunny Biotech Co. Ltd. (Shanghai, China). Orf assignments and their proposed function were accomplished by using the FramePlot 4.0beta [37] and Blast programs [38], respectively. 

### 3.3. Genomic Library Construction

*S. sanyensis* FMA genomic DNA was partially digested with *Sau*3AI, and fragments of 40–50 kb were recovered and dephosphorylated with CIAP and then ligated into SuperCos1 that was pretreated with *Xba*I, dephosphorylated and digested with *Bam*HI. The ligation product was packaged into lambda particles with the MaxPlax Lambda Packaging Extract (Epicenter, Madison, WI, USA), as per the manufacture’s instruction and plated on *E. coli* Top10. The titer of the primary library was about 2 × 10^5^ cfu per μg of DNA. 

### 3.4. Library Screening

According to the alignment result of RebC, StaC, AtmC and InkE (Figure 1), the FAD-dependent monooxygenases involved in ICZ ring biosynthesis, a pair of degenerate primers were designed as follows: *spcC*DFP, 5′-GABCTSGGCTGGAAGCTCGCCGC-3′/*spcC*DRP, 5′-GTCBCCGCGCCAGGCSACGTG-3′. The amplified PCR products were subcloned into pUM-T vector (Bioteke, Beijing, China) and then sequenced, revealing a DNA fragment with the size of 596 bp, according to which another pair of specific primers (*spcC*SFP, 5′-AGTTGCCGCCCGACATCC-3′/*spcC*SRP, 5′-GCCCGCTCGTACAGCTTGG-3′) was designed for library screening against 2500 colonies by PCR.

### 3.5. Gene Inactivation

Gene inactivation in *S. sanyensis* FMA was performed using the REDIRECT Technology, according to the literature protocol [34,35]. The amplified *aac(*3*)IV-oriT* resistance cassette from pIJ773 was transformed into *E. coli* BW25113/pIJ790/pWLI615 to replace an internal region of the target gene. Mutant cosmids pWLI621 (Δ*spcC*), pWLI622 (Δ*spcI*) and pWLI623 (Δ*spcR*) were constructed (Table S2, Supplementary Materials) and introduced into *S. sanyensis* FMA by conjugation from *E. coli* ET12567/pUZ8002, according to the reported procedure [36]. The desired mutants were selected by the apramycin-resistant and kanamycin-sensitive phenotype and were further confirmed by PCR (Table S3, Supplementary Materials). 

### 3.6. Heterologous Expression of the *spc* Gene Cluster in *S. coelicolor* M1152

*S. coelicolor* M1152 was used as the surrogate host for heterologous expression. A DNA fragment from pSET152AB was transformed into *E. coli* BW25113/pIJ790/pWLI615 to insert the *aac(3)IV-oriT -*φC31-attP/int into the neomycin resistance gene of SuperCos1. The resulting cosmid pWLI617 was passed through *E. coli* ET12567/pUZ8002 and then introduced into *S. coelicolor* M1152 via conjugation, according to the established procedure [36]. Apramycin-resistant exconjugants were selected to afford *S. coelicolor* M1152/pWLI617. Fermentations of *S. coelicolor* M1152/pWLI617 and *S. coelicolor* M1152 were performed under identical conditions as the wild-type *S. sanyensis* FMA and were analyzed for ICZs production by HPLC with the wild-type strain FMA as a positive control. 

### 3.7. Production and Analyses of ICZs in *S. sanyensis* FMA Strains

For the production of ICZs, both seed and production media consisted of 1.5% soybean meal, 0.5% yeast extract, 0.2% soluble starch, 0.2% peptone, 0.4% NaCl, 0.4% CaCO_3_ and 3.3% sea salt, pH 7.3 [4]. Spores of FMA strains were first inoculated into 50 mL of seed medium in a 250 mL flask and incubated at 28 °C, 220 rpm for 2 days. The resulting seed cultures were used to inoculate the production medium (5 mL into 50 mL of medium in a 250 mL flask for production analysis or 20 mL into 200 mL in a 1 L flask for isolation) and incubated at 28 °C, 220 rpm for another 5 days. The fermentation cultures were harvested by centrifugation, and the supernatant was extracted twice with an equal volume of ethyl acetate. The combined EtOAc extracts were concentrated *in vacuo* to afford a brown residue. The mycelia were extracted twice with acetone. The combined acetone extracts were concentrated *in vacuo* to afford the water phase. The resulting water phase was extracted twice with EtOAc. The combined EtOAc extracts were concentrated *in vacuo* to afford a brown residue. The above residues were dissolved in MeOH, combined, filtered through a 0.2 μm filter and subjected to HPLC. The HPLC system consisted of Agilent 1260 Infinity Quaternary pumps and a 1260 Infinity diode-array detector. Analytical HPLC was performed on an Eclipse C18 column (5 μm, 4.6 × 150 mm) developed with a linear gradient from 30% to 100% MeOH/H_2_O in 20 min, followed by an additional 10 min at 100% MeOH at flow rate of 1 mL/min and UV detection at 290 nm. Semi-preparative HPLC was conducted using an YMC-Pack ODS-A C18 column (5 μm, 120 nm, 250 × 10 mm). Samples were eluted with a linear gradient from 70% to 95% MeOH/H_2_O in 25 min, followed by 100% MeOH for 5 min at a flow rate of 2.0 mL/min and UV detection at 290 nm. The identities of STA, K252c and K252d produced by FMA strains were confirmed by MS and NMR analysis. LC-MS was carried out on Agilent 6430 Triple Quadrupole LC mass spectrometer. NMR data was recorded with a Bruker Avance 600 spectrometer.

### 3.8. Nucleotide Sequence Accession Number

The nucleotide sequence reported in this paper has been deposited in the GenBank database under accession number KC182794. 

## 4. Conclusions

In conclusion, we described the cloning, characterization and heterologous expression of the ICZ gene cluster from the marine-derived *S. sanyensis* FMA. Inactivation of 3 *spc* genes confirmed its identity. Although this cluster is highly homologous to the STA gene cluster from *Streptomyces* sp. TP-A0274, a different phenotype of the aminotransferase gene mutant was observed. The accumulation of both K252c and K252d in *spcI* mutant revealed the relaxed substrate specificity of the *N*-glycosyltransferase SpcG. In addition, this cluster was expressed in *S. coelicolor* M1152 with a comparable yield. The work reported here represents the first cloning and characterization of an ICZ gene cluster from a marine-derived actinomycete strain and would be useful for comprehensive elucidation of the biosynthetic mechanism of ICZ glycosides.

## Supplementary Material

**Table S1:** Bacteria and plasmids used in this study.

Strains or plasmids	Description	Reference or source
Strains		
*E. coli* Top10	Host strain of cosmid vector SuperCos1	Invitrogen
*E. coli* DH5*a*	Host strain for general cloning	Stratagene
*E. coli* ET12567/pUZ8002	Host strain for conjugation	[39]
*E. coli* BW25113/pIJ790	Host strain for PCR-targeting	[40]
*Strptomyces sanyensis* FMA	Wild type, ICZs producer	[4]
LIW601	*Δ spcC* inactivation mutant of *S. sanyensis* FMA	This study
LIW602	*Δ spcI* inactivation mutant of *S. sanyensis* FMA	This study
LIW603	*Δ spcR* inactivation mutant of *S. sanyensis* FMA	This study
*Strptomyces coelicolor* M1152/pWLI617	*Strptomyces coelicolor* M1152 carrying pWLI617	This study
Plasmids		
SuperCosI	Ap^r^, Km^r^, cosmid vector	Stratagene
pUM-T	Ap^r^, general cloning vector	Bioteke
pIJ773	Apr^r^, source of *acc(3)IV*-*oriT* cassette	[34]
pIJ790	Cm^r^, λ RED recombination plasmid	[34]
pWLI601	pUM-T cloned with the amplified FAD-dependent monooxygenase gene fragment	This study
pWLI611-615	Genomic library cosmids harboring *spc* biosynthetic genes from *S. sanyensis* FMA	This study
pWLI621	pWLI615 derivative where *spcC* was replaced with *acc(3)IV*-*oriT* cassette	This study
pWLI622	pWLI615 derivative where *spcI* was replaced with *acc(3)IV*-*oriT* cassette	This study
pWLI623	pWLI615 derivative where *spcR* was replaced with *acc(3)IV*-*oriT* cassette	This study
pWLI617	pWLI615 derivative which was equipped with *oriT* and φC31 attP/int	This study
pSET152AB	pSET152 derivative	[41]

**Table S2:** The primer pairs used for PCR-targeted mutagenesis^a^.

Gene	Primer pairs used for inactivation (5'→3')
*spcC*	*spcC*MF: GCCGCCCGACATCCTGGTGGACGGGCCCGAGGGCGACCGGattccggggatccgtcgacc
	*spcC*MR: CGCTCGTACAGCTTGGCGACCTCCTCGCCGCCCCCGCCGCGtgtaggctggagctgcttc
*spcI*	*spcI*MF: CATGACCACGCGAGTATGGGACTACCTGGCGGAGTACGAGattccggggatccgtcgacc
	*spcI*MR: CCTCACAGCGTCTCCAGCACCTCGCGCAGCGCGTGGACGACtgtaggctggagctgcttc
*spcR*	*spcR*MF: CATGGGTCCTCAGGTACGAGCGTTAGCACCGCTGCGCGGCattccggggatccgtcgacc
	*spcR*MR: CCTCAGGTCCGGAAGCGCACCAGAGCGGTGCGGGAGCGTATtgtaggctggagctgcttc

^a^ Underlined letters represent nucleotides homologous to the DNA regions internal to target genes.

**Table S3:** The primer pairs used for PCR confirmation of the mutants.

Gene	Primer pairs designed to verify the mutant strains (5'→3')	Length of fragment replaced	Length of desired PCR fragments	
			Wild type	Mutant
*spcC*	*spcC*CF: AGTTGCCGCCCGACATCC	321 bp	409 bp	1470 bp
	*spcC*CR: GCCCGCTCGTACAGCTTGG			
*spcI*	*spcI*CF: GACCACGCGAGTATGGGACTAC	1032 bp	1111 bp	1467 bp
	*spcI*CR: GCCTCACAGCGTCTCCAGCA			
*spcR*	*spcR*CF: GGTCCCGTCCCCTTCGACA	2883 bp	3001 bp	1540 bp
	*spcR*CR: GGCCTCAGGTCCGGAAGC			

**Table S4:** Assignments from 600MHz ^1^H NMR spectroscopy of STA in CDCl_3_^a^.

Position	δ_H_ (numbers of H, multiplicity ^b^, *J*, Hz)
1	7.30 (1H, d, 7.9)
2	7.48 (1H, dt, 7.7, 1.1)
3	7.36 (1H, dt, 7.5, 0.5)
4	9.41 (1H, d, 7.9)
6	6.21 (1H, brs)
7	5.03 (2H, AB, 16.1)
8	7.90 (1H, d, 7.7)
9	7.32 (1H, t, 7.5)
10	7.42 (1H, dt, 7.7,1.1)
11	7.93 (1H, d, 8.4)
3′	3.88 (1H, d,3.6)
4′	3.35 (1H, m)
5′	2.41 (1H, ddd, 14.9,5.7,3.7), 2.75 (1H, ddd, 14.9,4.0,1.2)
6′	6.57(1H, dd, 5.7,1.1)
2′-CH_3_	2.36 (1H, s)
3′-OCH_3_	3.41 (1H, s)
4′-NCH_3_	1.54 (1H, s)

^a^ δ_TMS_ = 0. ^b^ Multiplicity: s, singlet; d, doublet; dd, doublet of doublets; dt, doublet of triplets; ddd, doublet of doublet of doublets; m, multiplets; AB, AB quartet; brs, broad singlet.

**Table S5:** Assignments from 600MHz NMR spectroscopies of K-252c in DMSO ^a^.

Position	δ_H_(multiplicity ^b^, *J*, Hz)	δ_C_
1	7.70 (d, 8.1)	111.9
2	7.43 (dt, 7.7, 1.1)	125.5
3	7.22 (dt, 7.5, 0.8)	119.4
4	9.20(d, 7.9)	125.3
6	8.44 (brs)	/
7	4.96 (s)	45.7
8	8.04 (d, 7.7)	121.6
9	7.31 (dt, 7.5, 0.8)	120.2
10	7.48 (dt, 7.7,1.1)	125.3
11	7.77 (d, 8.1)	112.2
12	11.70(brs)	/
13	11.52(brs)	/

^a^ DMSO is used as internal reference. δ_H_ = 2.50, δ_C_ = 39.9. ^b^ Multiplicity: s, singlet; d, doublet; dt, doublet of triplets; brs, broad singlet.

**Table S6:** Assignments from 600MHz NMR spectroscopies of K-252d in DMSO ^a^.

Position	δ_H_(multiplicity ^b^, *J*, Hz)	δ_C_
1	7.69 (d, 8.5)	110.3
2	7.48 (m)	125.4
3	7.27 (dt, 7.5, 0.8)	119.7
4	9.45(d, 7.8)	125.8
4a	/	122.9
4c	/	118.8
5	/	172.4
6	8.53 (brs)	/
7	5.00 (AB,17.5)	45.6
7a	/	134.4
7b	/	115.4
7c	/	122.5
8	8.06 (d, 7.8)	121.5
9	7.31 (dt, 7.4, 0.9)	120.2
10	7.50 (m)	125.4
11	7.60 (d, 8.1)	111.8
11a	/	139.4
12	11.68 (brs)	/
12b	/	124.9
13a	/	140.5
1′	6.39 (d, 9.6)	77.3
2′	4.48 (m)	67.2
3′	4.17 (dd, 3.6, 5.9)	72.1
4′	4.04 (dd, 1.0, 3.6)	71.9
5′	4.47 (m)	76.7
6′	1.69 (d, 7.3)	15.8

^a^ DMSO is used as internal reference. δ_H_ = 2.50, δ_C_ = 39.9. ^b^ Multiplicity: s, singlet; d, doublet; dd, doublet of doublets; dt, doublet of triplets; m, multiplets; brs, broad singlet. /: there is no H or C at this position.
